# Defining Bronchial Asthma with Phosphoinositide 3-Kinase Delta Activation: Towards Endotype-Driven Management

**DOI:** 10.3390/ijms20143525

**Published:** 2019-07-18

**Authors:** Jae Seok Jeong, Jong Seung Kim, So Ri Kim, Yong Chul Lee

**Affiliations:** 1 Department of Internal Medicine, Research Center for Pulmonary Disorders, Chonbuk National University Medical School, Jeonju 54907, Korea; 2 Research Institute of Clinical Medicine of Chonbuk National University-Biomedical Research Institute of Chonbuk National University Hospital, Jeonju 54907, Korea; 3 Department of Otorhinolaryngology-Head and Neck Surgery, Chonbuk National University Medical School, Jeonju 54907, Korea

**Keywords:** bronchial asthma, endotype, precision medicine, phosphoinositide 3-kinase delta

## Abstract

Phosphoinositide 3-kinase (PI3K) pathways play a critical role in orchestrating the chronic inflammation and the structural changes of the airways in patients with asthma. Recently, a great deal of progress has been made in developing selective and effective PI3K-targeted therapies on the basis of a vast amount of studies on the roles of specific PI3K isoforms and fine-tuned modulators of PI3Ks in a particular disease context. In particular, the pivotal roles of delta isoform of class I PI3Ks (PI3K-δ) in CD4-positive type 2 helper T cells-dominant disorders such as asthma have been consistently reported since the early investigations. Furthermore, there has been great advancement in our knowledge of the implications of PI3K-δ in various facets of allergic inflammation. This has involved the airway epithelial interface, adaptive T and B cells, potent effector cells (eosinophils and neutrophils), and, more recently, subcellular organelles (endoplasmic reticulum and mitochondria) and cytoplasmic innate immune receptors such as NLRP3 inflammasome, all of which make this PI3K isoform an important druggable target for treating asthma. Defining subpopulations of asthma patients with PI3K-δ activation, namely PI3K-δ-driven asthma endotype, may therefore provide us with a novel framework for the treatment of the disease, particularly for corticosteroid-resistant severe form, an important unresolved aspect of the current asthma management. In this review, we specifically summarize the recent advancement of our knowledge on the critical roles of PI3K-δ in the pathogenesis of bronchial asthma.

## 1. Introduction

Bronchial asthma is a representative allergic inflammatory disorder of the airways, wherein a spectrum of respiratory symptoms including cough, wheezing, chest tightness, and shortness of breath present variably over time in association with chronic airway inflammation and airway hyperresponsiveness (AHR). Traditionally, the pathogenesis of chronic airway inflammation in asthma was generally understood to be a childhood-onset disease related to atopy/allergy. However, numerous recent clinical studies across human asthma cohorts in the United States and Europe have consistently demonstrated that the prevalence of atopy/allergy decreases in adult-onset and severe disease. They have also shown that asthma comprises diverse clinical and molecular phenotypes necessitating more precise and tailored treatment approaches according to causative pathobiologic mechanisms (i.e., endotype), particularly in the severe form of the disease [1]. In other words, asthma does not represent a single disease, rather a clinical syndrome in which multiple pathobiologic mechanisms may contribute to chronic airway inflammation, leading to similar clinical manifestations [2,3].

Recently, both advancement in our understanding of asthma pathogenesis and the clinical success of biologic therapies interfering with type 2 cytokine signaling related to interleukin (IL)-5, IL-4, and IL-13 in severe asthma patients [4,5] have led to the current dichotomy of type 2 and non-type 2 inflammation, which will improve our interpretation of the extremely heterogeneous nature of chronic inflammation in asthma. Moreover, molecular phenotyping of asthma into type 2 and non-type 2 is commonly used interchangeably with eosinophilic (i.e., cellular profiles demonstrate a significant number of eosinophils) and non-eosinophilic inflammation (i.e., inflammatory cell types may include neutrophils, mixed granulocyte inflammatory cells, or few inflammatory cells, also known as paucigranulocytic inflammation), respectively, on the basis of underlying inflammatory cellular profiles in sputum, airway, and/or blood from asthma patients [3]. However, there is a lack of available therapeutic agents for non-type 2 inflammation, which is estimated to contribute to approximately 50% of all asthma and is known to be frequently associated with corticosteroid (CS)-resistant severe airway inflammation. Furthermore, numerous possible pathobiologic mechanisms related to eosinophilic or non-eosinophilic inflammation have been proposed to exist either individually or to coexist with each other, giving further clinical heterogeneity both in type 2 and non-type 2 asthma [2,3]. These findings indicate that simple categorization of heterogeneous bronchial asthma into the dichotomy of type 2 and non-type 2 may be insufficient for developing novel therapeutic agents for bronchial asthma. In this regard, a pathobiologic mechanism that encompasses diverse physiological and pathological conditions involving various cell types may have the potential to integrate complex and heterogenous inflammation of bronchial asthma into a certain context as a novel endotype. Furthermore, this approach enables us to develop more precise and tailored treatment options for individual patients (i.e., precision medicine), particularly for a patient with severe asthma who has not responded well to the current maximal treatments.

Phosphoinositide 3-kinases (PI3Ks) are critical players in a myriad of cellular events and have been regarded as potential druggable targets for numerous human disorders [6]. In fact, throughout the intensive studies on the development of effective PI3K inhibitors, researchers have been confronted with potential dose-limiting and unpredicted adverse effects, partly owing to the importance of this pathway in maintaining general cell biology in nearly all cell types and to the nonselective inhibitory profiles of investigational drugs to various isoforms of PI3Ks simultaneously. Recently, tremendous progress has been made in developing selective and effective PI3K-targeted therapies for the treatment of cancer and other immune/inflammatory diseases on the basis of a vast amount of studies on the roles of specific PI3K isoforms and fine-tuned modulators of PI3Ks in a particular disease context.

In this review, we specifically summarize the recent advancement of our knowledge on the critical roles of the delta isoform of PI3Ks (PI3K-δ) in the pathogenesis of bronchial asthma, thereby suggesting a novel framework for the treatment of the PI3K-δ-driven asthma endotype.

## 2. Introduction to Class I PI3K and Its Isoforms

The PI3K pathway regulates pleiotropic function across diverse cell types in the context of both normal physiology and pathobiologic processes. Activation of class I PI3K, the most studied group, occurs downstream of a wide array of growth factor receptors and oncogenes, and it catalyzes the reversible phosphorylation of membrane inositol lipids at the D-3 position [i.e., converts phosphatidylinositol-4-5-bisphosphate (PtdIns-4,5-P_2_) into phosphatidylinositol-3-4-5-trisphosphate (PtdIns-3,4,5-P_3_)], which generates a lipid second messenger to recruit cytosolic signaling proteins having pleckstrin homology (PH) domains. A representative effector of PI3K is a serine/threonine kinase, AKT, and phosphorylation of AKT serves as a surrogate marker of class I PI3K activation. During this process, dual recruitment of AKT and its upstream activating kinase, phosphoinositide-dependent kinase-1 (PDK-1), to the plasma membrane and binding of both proteins to PtdIns-3,4,5-P_3_ via PH domains facilitate PDK-1-mediated phosphorylation of AKT. The activated form of AKT then phosphorylates numerous cytosolic substrates implicated in controlling nearly all aspects of cellular events, including cell growth and proliferation, metabolism, motility, and survival. Meanwhile, the activated PI3K pathway, in response to growth factor stimulation, should be rapidly repressed in normal cells to maintain cellular homeostasis. The tumor suppressor phosphatase and tensin homolog (PTEN) is the main endogenous negative regulator of PI3K in cells, which terminates signaling through dephosphorylation of PtdIns-3,4,5-P_3_ to PtdIns-4,5-P_2_. Initially, studies on the PI3K pathway were mainly focused on its role in cellular responses to growth factors and malignant transformation, which were followed by intensive research in the oncological field for over 30 years [6]. Indeed, researchers have found that the increased activity of PI3K-related pathways (e.g., activation of PI3K itself or loss of function of PTEN) is critically implicated in many human cancers [7]. In addition, the PI3K pathway is closely associated with other critical regulators of cell cycle progression and proliferation including KRAS [8], thereby contributing to tumorigenesis in the cell. At the same time, in parallel to PI3K research by cancer biologists, studies using numerous PI3K-targeted therapies have revealed that the PI3K pathway contributes to a broad spectrum of human diseases including immune/inflammatory diseases, endocrine diseases, and cardiovascular diseases. Experimental results from mouse genetic models with deletion or mutation of certain PI3K-encoding genes have further facilitated research on the unique and the redundant roles of PI3K isoforms in mammalian cells and tissues [6,9].

PI3Ks are heterodimers composed of a p110 catalytic subunit and a regulatory subunit, and mammals express four class I catalytic isoforms. Class IA PI3K is divided into three isoforms according to their p110 catalytic subunit, namely p110α, p110β, and p110δ, while p110γ solely constitutes class IB PI3K. Catalytic subunits of class IA PI3K associate with regulatory subunits (i.e., p85, p55, or p50) whose SH2 domains bind to phosphotyrosyl residues on growth factor receptors or adaptor proteins, whereas the catalytic subunit of class IB PI3K associates with regulatory subunits (i.e., p101 or p84/p87) that allow PI3K activation by the βγ subunits of G protein-coupled receptors [10]. Expression of p110α and p110β protein is ubiquitous and is required for embryonic development and cell proliferation. Therefore, genetic knockdown of these isoforms leads to embryonic lethality [11,12], and therapeutic targeting of those isoforms for certain disorders may be nonselective enough to cause systemic adverse effects. However, expression of p110δ and p110γ isoforms is predominantly restricted in hematogenous immune and inflammatory cells, and these isoforms are the dominant class I PI3K isoforms in leukocytes, which implies their critical involvement in various facets of the innate and adaptive immune system and their potential as druggable targets for developing PI3K-targeted therapies. As for pulmonary disorders, the p110δ isoform has been shown to play important roles in the inflammatory process of bronchial asthma and chronic obstructive pulmonary disease, especially in mediating CS resistance [13,14,15,16]. Furthermore, a recently described immune deficiency in humans, the activated PI3K-δ syndrome (APDS) (e.g., several point mutations occur in the p110δ catalytic domain, leading to exaggerated PI3K-δ signaling), clearly demonstrates the clinical outcomes associated with aberrant activation of PI3K-δ, including recurrent respiratory infections, airway damage and the extremely high incidence of bronchiectasis, and dysfunctional T and B lymphocytes, all of which highlight the critical role of this isoform, particularly in the respiratory system [17,18].

## 3. Roles of PI3K-δ in Type 2 Inflammation

In the view of classic allergic airway inflammation, adaptive CD4-positive type 2 helper T cells (TH2 cells), which are stimulated by dendritic cells (DCs), orchestrate eosinophilic airway inflammation and AHR through producing type 2 cytokines [2,3]. Among them, IL-4 is necessary for the class switching of the immunoglobulins to immunoglobulin E (Ig-E) by B cells and the subsequent generation of an immediate allergic reaction mediated by mast cells and basophils via Ig-E cross-linking. The presence of allergen-specific Ig-E is the hallmark of the adaptive TH2 response related to atopy/allergy. IL-13 is thought to be important in airway mucin production and maintenance of AHR. In particular, IL-5 is closely implicated in eosinophilia in lung tissue and blood and is regarded as one of most potent inflammatory mediators in the induction and the maintenance of eosinophilic inflammation, making this cytokine an important druggable target for treating severe asthma [4]. Clinically, TH2 asthma is known to respond well to corticosteroids (CS) and represents a common pathobiologic mechanism among many allergic diseases, including allergic rhinitis and atopic dermatitis. Recently, another eosinophilic type 2 inflammation, which is controlled in a T-cell independent manner, was characterized. In this molecular subphenotype of type 2 inflammation, namely T2 inflammation, epithelial cells play a key role in the initiation of eosinophilic inflammation by producing epithelium-derived cytokines [i.e., alarmins including IL-25, IL-33, and thymic stromal lymphopoietin (TSLP)] in response to epithelial activation from epithelial injury and activation of pattern recognition receptors (PRRs) abundantly expressed in the cellular surface [19]. This process leads to the activation of type 2 innate lymphoid cells (ILC2 cells) devoid of antigen-specific receptors and subsequent production of type 2 cytokines including IL-5 and IL-13. T2 inflammation is not mediated in an antigen-specific manner like adaptive immunity and is thus called an innate-like response, which in part may explain why—very early after allergen exposure or single allergen exposure—the production of IL-5 and IL-13 necessary for eosinophilic type 2 inflammation can be induced [20]. In this context, increasing evidence indicates that T2 eosinophilic inflammation is relatively resistant to CS and is crucially implicated in the pathogenesis of acute asthma exacerbation and severe asthma [21].

**Figure 1:**
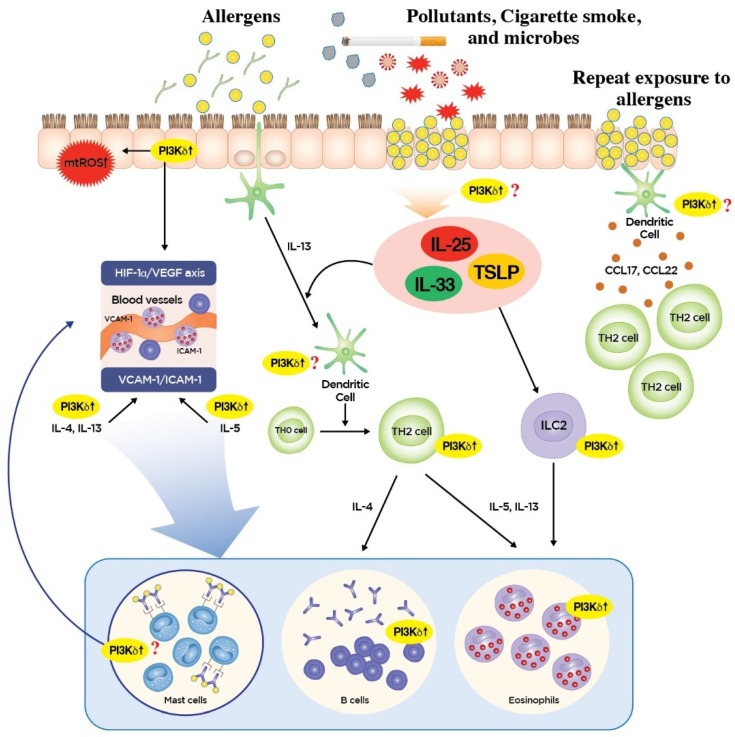
Roles of phosphoinositide 3-kinase-δ (PI3K-δ) in type 2 inflammation of bronchial asthma. PI3K-δ may contribute to various aspects of allergic and non-allergic eosinophilic airway inflammation involving diverse cell types. In the airway epithelial interface, exposure to aeroallergens such as fungus (e.g., *Aspergillus fumigatus*), air pollutants, cigarette smoke, and microbes leads to the activation of epithelial PI3K-δ, which is critically implicated in the generation of mitochondrial reactive oxygen species (mtROS), activation of hypoxia-inducible factor (HIF)-1α-vascular endothelial growth factor (VEGF) axis, and possibly the release of alarmins (e.g., IL-33, IL-25, and TSLP) in the airway epithelium (our preliminary data). Airway epithelial generation of mtROS has been known to be essential for CD4–positive type 2 helper T (TH2) cell-associated eosinophilic inflammation in airways in part through modulation of cytoplasmic nucleotide-binding domain, leucine-rich repeat-containing family protein-3 (NRLP3) inflammasome (not shown here). Epithelial activation of HIF-1α-VEGF axis regulates vasodilation, vascular permeability, and subsequent protein extravasation, all of which are cardinal features of airway inflammation of asthma. Furthermore, epithelium-derived alarmins enhance both the dendritic cell (DC) migration to draining lymph nodes, wherein the adaptive TH2 immunity develops and the type 2 innate lymphoid (ILC2) cells activate, thereby producing type 2 cytokines essential for eosinophilic type 2 inflammation. IL-5 is one of the most important cytokines in eosinophilic lung inflammation. In addition, IL-4 and IL-13 prime vessel wall for inflammatory cell recruitments and IL-5 regulates transmigration of eosinophils, which occur through regulating vascular cell adhesion molecule-1 (VCAM)-1 and intercellular adhesion molecule-1 (ICAM-1), respectively. These type 2 cytokines and the activation of PI3K-δ are also essential for the optimal effector function in various cell types including eosinophils, Ig-E-producing B cells, and possibly mast cells. In particular, IL-33-mediated activation of TH2 and ILC2 cells is known to be dependent on PI3K-δ activity in both cell types. When the lungs are repeatedly exposed to the same allergens, monocytic DCs may recruitment of effector TH2 cells through production of CCL17 and CCL22, chemokines responsible for the allergen-induced recruitment and reactivation of TH2 cells. Processes that are known to be or that could be dependent on the activity of PI3K-δ are indicated.

Although the relative contribution of TH2 versus T2 inflammation in eosinophilic inflammation of asthma is unknown and may vary according to the type of allergenic or antigenic stimuli exposed to lungs, PI3K-δ may contribute to various aspects of allergic and non-allergic eosinophilic airway inflammation involving diverse cell types given the preferential expression of PI3K-δ in immune/inflammatory and structural cells such as airway epithelium (Figure 1).

Firstly, PI3K-δ may potently influence the TH2/T2 immune response in the airway epithelial interface. In fact, airway epithelium is the first cell layer to encounter allergens and is an essential controller of innate and adaptive immune responses in allergic lung inflammatory disorders. In particular, airway epithelial release of alarmins can result from PRR activation such as toll-like receptor (TLR) 4 expressed on airway epithelial cells [19,22]. Then, resultant cytokine milieus can activate dendritic cells, ILC2 cells, and basophils, thereby promoting migration of DCs to the draining lymph nodes with the subsequent induction of adaptive immune response [19]. Our preliminary data demonstrate that blockade of PI3K-δ using a potent specific inhibitor significantly reduced the respiratory allergen-induced elevation of IL-33 protein in lungs of mice. Furthermore, IL-33-mediated activation of TH2 and ILC2 cells is also known to be dependent on PI3K-δ activity in both cell types [23]. Recently, airway epithelial PI3K-δ was also reported to be a crucial modulator of mitochondrial reactive oxygen species (mtROS), and these are known to be essential for TH2 cell-associated eosinophilic inflammation in airways [24] and nucleotide-binding domain, leucine-rich repeat-containing family protein-3 (NRLP3) inflammasome. NLRP3 inflammasome is one of the cytoplasmic PRRs necessary for the generation of a potent inflammatory mediator IL-1β, thereby it is critically implicated in fungus-induced CS-resistant eosinophilic lung inflammation [16]. In addition, PI3K-δ in airway epithelial cells is involved in the pathogenesis of allergic lung disorders through modulating the release of pro-inflammatory mediators such as hypoxia-inducible factor (HIF)-1α and vascular endothelial growth factor (VEGF), all of which have been reported to play a crucial role in mediating vasodilation, increased permeability, and subsequent protein extravasation in allergic airway disease [25,26]. However, as for the role of PI3K-δ in DC function in the context of epithelial cell–DC interaction, there are insufficient data supporting its role in antigen presentation given that mRNAs of PI3K-δ are expressed predominantly in plasmacytoid DCs, producing large amounts of type I interferon (IFN) mediating anti-viral immunity, and not in conventional DCs, the main subset for antigen presentation [27]. Instead, PI3K-δ in DC has been reported to be a homeostatic regulator of TLR4 signaling by inducing internalization of TLR4 from the plasma membrane, thereby attenuating pro-inflammatory cytokine secretion against endotoxins in this cell type [28]. Recently, in a clinical phase 1 study, treatment with an oral PI3K-δ inhibitor, idelalisib (also known as CAL-101), in patients with allergic rhinitis significantly reduced plasma CCL17 and CCL22, chemokines secreted mainly from DCs responsible for the allergen-induced recruitment of TH2 lymphocytes, suggesting an additional role of PI3K-δ in the regulation of DC function [29]. The roles of PI3K-δ in diverse functional aspects and various subsets of DCs need to be further defined in the future (Figure 1).

Secondly, PI3K-δ has been reported to play an important role in the adaptive immune response involving T and B cells [30,31], thereby potently influencing adaptive TH2 cell-mediated inflammation and associated eosinophilic inflammation. In the initial period of research, investigation of the specific roles of each class IA PI3K isoform in adaptive immune cells was not possible, as most studies were performed in mice bearing genetic deletions of the regulatory subunits, which are common to overall class IA PI3K [32]. Subsequent studies developed novel tools to evaluate the specific role of PI3K-δ in T and B cells and revealed that kinase-inactivating mutation (D910A) of the p110δ isoform did not lead to significant abnormality of T cell development; however, impairment of T cell receptor-mediated cell proliferation was observed [30]. Moreover, by using the same p110δ^D910A/D910A^ T cells, clonal expansion and differentiation of helper T cells (TH cells) were shown to be controlled by PI3K-δ [33]. In addition, activation of PI3K-δ under the engagement of T cell receptor with antigen is important for T cell localization to antigenic tissue [34]. In terms of the effector function of T cells, blockade of PI3K-δ using a potent inhibitor significantly reduced T cell receptor-induced PI3K signaling and cytokine production by both naïve and memory human T cells, highlighting the potential of PI3K-δ blockade in the treatment of T cell-mediated autoimmune and inflammatory diseases [35]. However, there seems to be a certain degree of functional overlap between different isoforms of PI3Ks in individual aspects of T cell function [36]. PI3K-δ also plays an essential role in B cell development, proliferation, differentiation, antibody class switching, and production [30,37,38]. Compared to p110δ^D910A/D910A^ T cells, in which antigen-induced proliferation can be overcome occasionally through strong co-stimulatory signaling [30], p110δ^−/−^ B cells have major functional defects in activation and antibody responses [39]. These findings mean that small molecule inhibitors of PI3K-δ need to be actively investigated to treat B cell-mediated hematologic malignancies and autoimmune inflammation [40]. However, in the allergic context, genetic and pharmacologic inhibition of PI3K-δ lead to enhanced immunoglobulin class switching to Ig-E in B cells in vivo despite decreased type 2 cytokine and Ig-G production, which emphasizes the homeostatic regulatory function of PI3K-δ in restraining Ig-E production [41]. Taken together, the roles of PI3K-δ in adaptive immunity involving T and B cells may be much more complicated than we anticipated (Figure 1).

Lastly, PI3K-δ has been shown to be implicated in diverse effector cell types of the TH2/T2 immune response, mainly eosinophils. IL-5 is the most important cytokine for maturation of eosinophils in bone marrow, and eosinophil growth, differentiation, and tissue survival are highly dependent on IL-5. For example, once the eosinophil has entered the target tissue from the blood, the usual life-span of this cell type (ranging from two to five days) can be increased up to 14 days (in vitro) under the influence of various cytokines, including IL-5. Previous studies showed that PI3K-δ blockade using IC87114, a potent selective inhibitor of PI3K-δ, significantly lowered the allergen-induced increased levels of IL-5 protein in the lung [15,42]. In addition, the selective tissue recruitment of eosinophils involves numerous chemo-attractants and adhesion molecules. The most potent chemo-attractants include CC chemokines (e.g., CCL11/eotaxin-1, CCL5/RANTES), lipid mediators [e.g., platelet-activating factors (PAF); leukotriene D4 (LTD4) metabolized from LTC4), and complements (e.g., CD5a)]. Among them, CC chemokines seem to be essential for inducing the migration of eosinophils to the inflamed tissues, because this cell type prominently and selectively expresses CCR3, which binds many chemokines, including eotaxin and RANTES. Furthermore, the inflammatory milieus of surrounding tissues involve cytokines and chemokines, which together modulate the transmigration of eosinophils into tissue. In particular, IL-4 and IL-13 have been reported to be involved in upregulating eosinophil adhesion through induction of vascular cell adhesion molecule (VCAM)-1 on endothelial cells [43]. IL-5 also enhances adhesion to the endothelium/transmigration of eosinophils through intercellular adhesion molecule (ICAM)-1. Previous reports showed that inhibition of PI3K-δ using IC87114 significantly lowered the allergen-induced increases in LTC4, eotaxin, RANTES, ICAM-1, VCAM-1, IL-4, IL-5, and IL-13 in the lung [42]. Similarly, adhesion of the PI3K-δ inhibitor-treated eosinophils to VCAM-1 and ICAM-1 was significantly attenuated, and pre-incubation of eosinophils with this PI3K-δ inhibitor significantly blocked eotaxin-1-induced migration and prevented changes in cell morphology, although there was no alteration in CCR3 expression [44]. The effector functions of eosinophils are mainly attained through the release of diverse eosinophil-derived mediators (e.g., granular proteins, lipid mediators, cytokines/chemokines, enzymes, growth factors, and oxidative products). Eosinophils express a wide range of cell surface receptors (e.g., receptors for cytokines/chemokines, immunoglobulins, complements, and lipid mediators and PRRs), and receptor engagement induces phenotypic and functional changes of eosinophils, including enhanced cell survival, augmented cell adhesion, and release of eosinophil-derived proinflammatory mediators. For example, exposure of eosinophils to various cytokines such as type 2 cytokines (IL-5, IL-4, and IL-13) activates lipid mediators and ROS generation, phagocytosis, and helminthotoxic activity. IL-33 has also been reported to activate eosinophilic effector functions as potently and effectively as IL-5 [45,46]. In particular, IL-33 has been reported to induce PI3K activation in human eosinophils, thereby promoting interaction with structural cells and release of pro-inflammatory cytokines and chemokines [47]. In addition, as outlined above, blockade of PI3K-δ significantly reduced the respiratory allergen-induced elevation of IL-33 protein in the lung (preliminary data), suggesting the potential implication of the PI3K-δ pathway in the modulation of eosinophilic effector functions. Meanwhile, as one of the innate immune cells, human eosinophils express various PRRs, including Toll-like receptors (TLRs 1 to 5, 7, and 9), C-type lectin receptors (Dectin-1), and nucleotide-binding oligomerization domain (NOD)-like receptors (NLRs; NOD1 and NOD2) [48]. PRRs activate downstream signaling cascades involving nuclear factor (NF)-κB that is crucial for subsequent generation of ROS and pro-inflammatory cytokines. Previous studies demonstrated that PI3K-δ can be closely associated with PRR signaling pathways such as TLRs and NLRs [16,28,49]. Our previous reports also showed that inhibition of PI3K-δ significantly reduced the respiratory allergen-induced increases of NF-κB nuclear translocation and generation of ROS, particularly mtROS [15,16] (Figure 1).

## 4. Roles of PI3K-δ in Non-Type 2 Inflammation

Previous studies demonstrated that non-type 2 asthma generally represents predominantly neutrophilic or paucigranulocytic inflammation with a relatively low TH2/T2 inflammatory profile. Clinically, this subtype of asthma is frequently associated with late-onset and CS-resistant disease and lower lung function. Increasing evidence indicates that pathobiologic mechanisms promoting neutrophilic inflammation such as TH1 (type 1) and TH17 (type 17) are critically involved in the development of non-type 2 asthma. For examples, IL-17 is a potent inducer of CS-resistant neutrophilic inflammation [50], and expression of type 17 cytokines such as IL-17A and IL-17F has been shown to correlate with disease severity in asthmatic airway tissue [51,52]. Moreover, the TH1/IFN-γ pathway and the tumor necrosis factor (TNF) were also reported to be implicated in neutrophilic inflammation, in part through being associated with TH17 inflammation. Patients having more severe asthma tended to possess more IFN-γ-positive and IL-17A-positive CD4-positive T cells in bronchoalveolar lavage (BAL) fluids [53], and there was an inverse correlation between the numbers of TH1-enriched CD4-positive T cells in BAL cells and the percent predicted forced expiratory volume in 1 s (FEV_1_) in severe asthma patients [54]. Moreover, TNFα neutralization significantly ameliorated TH17-mediated neutrophil infiltration into the lung in the adoptive TH17 cell transfer, ovalbumin-challenged murine model [55]. Interestingly, an unbiased molecular approach to analyze bronchial epithelial cell gene expression revealed a subset of severe asthma patients with higher expression of genes related to both IFN-γ and TNFα pathways [56]. Furthermore, a recent novel study using sputum cell transcriptomics from moderate-to-severe asthmatic subjects defined a unique transcriptome-associated cluster (TAC) characterized by predominant neutrophilic inflammation with IFN and TNF superfamily upregulation and high expression of damage-associated molecular patterns (DAMPs) and inflammasome-associated genes [57]. These findings highlight the fact that common cellular events relevant to the activation of IL-17, IFN-γ, and TNFα pathways may underlie the neutrophilic subphenotype of non-type 2 inflammation. In this respect, activation of innate immune signaling pathways, which frequently occurs in association with exposure to microbes (viruses, bacteria, and fungi) and injury (infection, air pollutants, cigarette smoke, and oxidative stress) in airway epithelial interface, has received particular attention as an important molecular mechanism for non-type 2 inflammation of airways [1,3].

**Figure 2:**
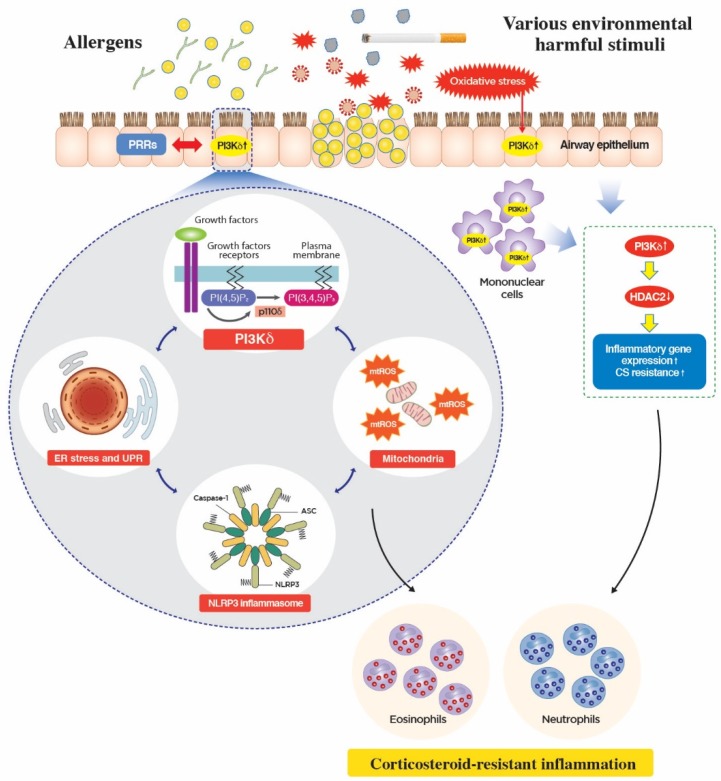
Mechanism of action of phosphoinositide 3-kinase-δ (PI3K-δ) in corticosteroid (CS)-resistant lung inflammation. Airway epithelial cell express pattern-recognition receptors (PRRs) that can be activated directly by allergens such as fungus. PI3K-δ can be closely associated with PRR signaling pathways, and the activation of PI3K-δ in airway epithelium results in oxidative stress, mainly from mitochondria (mitochondrial reactive oxygen species; mtROS). Airway epithelial activation of PI3K-δ has also been known to regulate endoplasmic reticulum (ER) stress and NLRP3 inflammasome, both of which are well-known inducers of CS resistant lung inflammation, partly through modulation of mtROS. These PI3K-δ-associated cytoplasmic molecular platforms involving ER, mitochondria, and NLRP3 inflammasome represent a novel mechanism for the involvement of PI3K-δ in the pathogenesis of CS-resistant severe eosinophilic inflammation. Meanwhile, in response to a wide array of harmful stimuli (e.g., exposure to air pollutants, cigarette smoke, and direct injuries), PI3K-δ can be closely associated with oxidative stress, especially in the airway epithelial interface. Subsequent activation of PI3K-δ in airway epithelial cells and mononuclear cells leads to the reduction of histone deacetylase 2 (HDAC2), a key molecule in the suppression of pro-inflammatory cytokine production in these cells, thereby inducing CS-resistant neutrophilic inflammation and further oxidative stress in the lung. IL-17 has been reported to reduce HDAC activity and CS sensitivity in epithelial cells (not shown here).

Although the pivotal roles of class I PI3Ks (specifically PI3K-δ in TH2-dominant disorders) have been consistently reported since the early investigational approaches [42,58,59,60], whether PI3K-δ plays a role in T cell polarization, particularly into TH1/TH17 and the related effector function, is controversial. Inhibition of overall class IA PI3K using in vivo protein transduction of dominant-negative regulatory subunit, p85α, in the allergic lung inflammation murine model did not influence TH1/IFN-γ responses and only regulated TH2 cytokine production (IL-4 and IL-5) [61]. However, a kinase-inactivating mutation of PI3K-δ (p110δ-D910A), particularly in hemopoietic cells, resulted in robust TH1-skewed responses such as increased production of IFN-γ and CXCL10 during the primary response to ovalbumin, which in part seemed to be associated with the lack of regulatory IL-10 production [62], while type 2 cytokine responses were substantially decreased in mice [60]. A similar phenomenon was observed in a study on the role of PI3K-δ in macrophages in the development of TH1/TH17 in microbiota-dependent colitis [63]. However, clonal expansion and differentiation of both TH1 and TH2 lineages were shown to be impaired in T cells harboring p110δ-D910A [33], and blockade of PI3K-δ effectively reduced cytokine production (IL-17, IFN-γ, Type 2 cytokines, and TNFα) from multiple T helper cell types including TH1, TH2, and TH17 [64], and naïve/memory T cells [35] from mice and human. It is assumed that these conflicting data may be partly explained by the difference in the timing of the modification of PI3K-δ activity (i.e., PI3K-δ blockade before or after the differentiation of TH cells) in various experimental settings. Furthermore, particularly in terms of the role of PI3K-δ in the effector function of various immune cells, inhibition of PI3K-δ has been shown to effectively suppress TH1/TH17 cytokines and TNFα in numerous pathologic conditions, including allergic airway disease [42,65], autoimmune and inflammatory arthritis/dermatitis [35,66], and neuroinflammation associated with ischemia/reperfusion [67], which involves diverse cell types (e.g., naïve/memory T cells, various TH cells, IL-17 producing TCRγδ T cells, and macrophages) crucial for neutrophilic inflammation [68]. As for neutrophilic inflammation, per se, PI3K has been known to influence several key functional aspects including chemotaxis, oxidative burst, and survival of neutrophils [14]. In particular, the crucial roles of PI3K-δ in the regulation of neutrophil trafficking and directional movement [69,70] and effector function such as oxidative burst [71] have been demonstrated. Furthermore, in response to a wide array of harmful stimuli (e.g., exposure to air pollutants, cigarette smoke, and injuries), PI3K-δ can be closely associated with oxidative stress, especially in the airway epithelial interface [16]. Activation of PI3K-δ in this interface (e.g., PI3K-δ activation in airway epithelial cells and mononuclear cells) can lead to the reduction of histone deacetylase 2 (HDAC2), which is a key molecule in the suppression of pro-inflammatory cytokine production in these cell types [72], impairment of CS responsiveness [13], increased inflammatory cell infiltration including neutrophils and subsequent production of pro-inflammatory cytokines, and oxidative stress. All of these processes may further perpetuate CS-resistant neutrophilic lung inflammation [73,74]. In fact, this particular role of PI3K-δ in mediating CS-resistant lung inflammation has received much attention in the development of new treatments for chronic obstructive pulmonary disease (COPD), a disorder believed to represent the neutrophil-dominant inflammatory process. Given the considerable overlap between severe asthma and COPD in pathobiology and the aforementioned critical roles in inducing and maintaining both eosinophilic and neutrophilic lung inflammation (Figure 2), the therapeutic strategy of modulating PI3K-δ activity in various immune cells may have the potential for treating allergic lung inflammation, particularly severe asthma, which is an extremely heterogeneous inflammatory condition comprising variable degrees of the TH1, the TH17, and the TH2 immune process [2,75].

## 5. Novel Mechanism of Action of PI3K-δ in CS-Resistant Lung Inflammation

In asthmatic chronic airway inflammation, diverse endotypes possessing unique pathobiologic mechanisms have been proposed to exist [3,76]. Moreover, differences in the extent of the relative contribution of numerous cell types implicated in innate/adaptive immune response and/or different types of aforementioned TH cell-related inflammation render pulmonary inflammation in each endotype more complex and unpredictable with regard to the therapeutic response to the conventional agents and clinical outcomes, which lead to the extreme clinical heterogeneity of asthma [2]. In this context, novel pathobiologic mechanisms critically implicated in general cell physiology across broad cell types have recently received considerable attention in this field of research, particularly the implications of functional disturbances in subcellular organelles such as endoplasmic reticulum (ER) and mitochondria in the pathogenesis of bronchial asthma [2,77,78]. Indeed, therapeutic restoration of functionalities in organelles may be ideal for drug development in asthma research, as this approach is less harmful and more physiologic. Interestingly, several crucial cellular mediators of inflammation have been reported to be interconnected to ER- and mitochondria-associated molecular networks, all of which are increasingly regarded as key inducers of CS-resistant inflammation in the lungs.

At the airway epithelial interface, environmental exposure to a myriad of potentially harmful extrinsic and/or intrinsic factors results in the activation of diverse cell types, particularly airway epithelium, through numerous cell surface receptors and direct cellular injury. Although the exact upstream modulators have not yet been clearly defined, being located in the proximity of various receptors (e.g., growth factors and cytokines receptors) and oxidative stress, PI3K-δ is activated to interact with ER- and mitochondria-associated molecular platforms. Previous studies demonstrated that the PI3K pathway can be closely related to ER stress through numerous mechanisms. For example, one report showed that regulatory subunits of PI3K, p85α, and p85β can interact with a crucial downstream effector pathway of ER stress, XBP-1 [79]. Moreover, another essential mediator of the ER stress pathway, PERK, can be associated with PI3K through regulating cellular localization of PTEN, one of the negative regulators of the PI3K pathway [80]. Furthermore, considering that close interrelationships exist between ER and mitochondria [81,82], the PI3K pathway may be linked to mitochondria in various aspects. In particular, mitochondria are an important source of cellular oxidative stress [83,84], and oxidative stress has been known to participate in diverse pathobiologic processes through modulation of the PI3K pathway in lungs [85]. In fact, the exact roles of the PI3K pathway in the modulation of oxidative stress are difficult to understand and considerably vary according to the physiologic/pathologic context and the experimental settings. For instance, a beneficial role of the PI3K/AKT pathway in generating antioxidant through inducing the nuclear factor erythroid 2-related factor 2-dependent antioxidant pathway in cells under oxidative injury, which was investigated using pan PI3K inhibitors (e.g., wortmannin and LY294002), has been reported [86]. However, inhibition of PI3K-δ using IC87114 or RNA interference remarkably improved fungus-induced CS-resistant eosinophilic lung inflammation through modulation of fungus-induced ER stress and associated cellular oxidative stress from mitochondria, particularly in airway epithelium [15]. These findings highlight the important role of cellular interconnection between PI3K-δ and subcellular organelles in inducing CS-resistant severe allergic lung inflammation, given that oxidative stress is a well-known inducer of CS resistance in the lung [87], and the ER stress pathway and PI3K-δ are also implicated in CS resistance partly through an oxidative stress-dependent manner [13,88,89]. More recently, another critical player in neutrophil-dominant CS-resistant inflammation in the lung, NLRP3 inflammasome [90,91], a cytoplasmic PRR responsible for producing potent pro-inflammatory IL-1β, was shown to be regulated by PI3K-δ partly through mtROS in airway epithelium, thereby also playing a key role in inducing CS-resistant eosinophilic lung inflammation [16]. Therefore, these PI3K-δ-associated cytoplasmic molecular platforms involving ER, mitochondria, and NLRP3 inflammasome represent a novel mechanism for the involvement of PI3K-δ in the pathogenesis of bronchial asthma, particularly for the severe form of the disease. Consistent with these findings, recent clinical data from a large European severe asthma cohort, U-BIOPRED, which aimed to stratify severe refractory asthma patients using a systematic biology approach such as sputum transcriptomic data [57], emphasized the role of PI3K-δ-associated mitochondria and inflammasome pathways as possible important endotypes of severe asthma (Figure 2).

## 6. PI3K-δ Inhibitors in Clinical Trials

On the basis of the aforementioned crucial roles of PI3K-δ in modulating various facets of lung inflammation, PI3K-δ is increasingly regarded as one of novel therapeutic targets in asthma and COPD [92]. However, given the unfavorable pharmacologic profiles of pan inhibitors of class I PI3K (e.g., wortmannin and LY294002) in preclinical studies that preclude their clinical use, researchers have focused on the optimization of lead compounds in regard to the isoform selectivity and the administration route in the development of effective PI3K-δ inhibitors for respiratory disorders. In this context, a novel inhaled PI3K-δ inhibitor, GSK2269557, was recently developed [93] and evaluated in several phase 2 clinical trials. Interestingly, considering the crucial involvement of PI3K-δ in mediating CS-resistant lung inflammation (Figure 2), those clinical studies were evaluated mainly among patients suffering from acute moderate to severe exacerbation of COPD (NCT02522299, NCT03345407, and NCT02294734) and persistent, uncontrolled asthma (NCT02567708). Some of the results are currently available (https://clinicaltrials.gov). In the view of side effect profiles, there was no remarkable difference regarding the incidence of serious adverse events from interfering the physiologic PI3K-δ activation in human immune system, including serious infections, neutropenia, skin rashes, gastrointestinal disorders (e.g., diarrhea and colitis), pneumonitis/organizing pneumonia, and hepatic impairment, all of which have been reported on the treatment with idelalisib, an oral PI3K-δ inhibitor approved for the treatment of multiple hematologic malignancies by the US Food and Drug Administration (FDA) and the European Medicines Agency (EMA) in 2014 [40,94], between two arms during the 12 week treatment period. Moreover, several phase I or phase 2 clinical trials have been performed using dual p110δ/p110γ inhibitors, including IPI-145 (phase 2a involving mild asthma subjects; NCT01653756) and RV-1729 (phase 1 involving healthy volunteers; NCT01813084 and NCT02140320) [95].

However, more precisely, clinical effectiveness and toxicity profiles of targeting respiratory PI3K-δ should be investigated in an optimal patient population possessing PI3K-δ-driven CS-resistant refractory endotype of the disease verified by proper biomarkers. In fact, there is still no validated biomarker that specifically indicates PI3K-δ activation in vivo. Interestingly, our preliminary data suggest that the catalytic subunit of PI3K-δ (p110δ) can be effectively detected through an electron microscope in the extracellular compartment of peripheral blood samples as well as the intracellular compartments of various inflammatory cells in mice. Further studies on the development of an optimal biomarker for the identification of the PI3K-δ-driven endotype of chronic respiratory diseases by using bloods, urine, and airway specimens including sputum from patients should be performed in the near future.

## 7. Conclusions

Since the initial discovery of the enzyme, the PI3K pathway has become one of the major fields of research for the development of novel therapies for the treatment of cancer and other immune/inflammatory diseases. During this process, the development of PI3K inhibitors has been challenged by the many unpredicted roles of this pathway implicated in normal cell function and in disease. However, along with the intensive studies on the role of specific isoforms of PI3Ks in various experimental systems and the results from oncology clinical trials in which many PI3K-targeted therapies have been evaluated, our knowledge about this pathway has been widened to apply specific isoforms of PI3Ks for therapeutic purposes. This has resulted in the recent regulatory approval of idelalisib for the treatment of certain types of hematologic malignancies [40,94]. In particular, for the respiratory system, the critical roles of PI3K-δ, a dominant isoform in inflammatory cells such as leukocytes, have been consistently reported, given that the lung is equipped with abundant blood vessels (i.e., a propensity for the recruitment of diverse hematogenous inflammatory cells into the lungs) and is continuously exposed to external environments (i.e., activation of PRRs and growth factor or cytokine receptors, all of which occur upstream of PI3K in various cell types). Furthermore, since the initial report on the crucial implication of PI3K-δ in the pathogenesis of bronchial asthma [42], there has been great advancement in our knowledge of the implications of PI3K-δ in various facets of allergic lung inflammation. This has involved the airway epithelial interface, adaptive T and B cells, potent effector cells including eosinophils and neutrophils, and, more recently, subcellular organelles (ER, mitochondria) and cytoplasmic innate immune receptors such as NLRP3 inflammasome. Importantly, activation of PI3K-δ is closely associated with a severe end spectrum of allergic inflammation, CS-resistant lung inflammation, which is an important, unresolved aspect of the current asthma management. Therefore, we suggest that PI3K-δ-driven asthma may represent an important endotype of asthma pathogenesis. Meanwhile, an important, unanswered question is how we can select optimal patient subpopulations in which therapeutic efficacy of PI3K-δ blockade will be maximized without serious adverse events related to drugs. Currently, we are devoid of reliable biomarkers indicative of PI3K-δ activation in vivo; therefore, bloods, urine, and airway specimens including sputum from asthma patients or, more specifically, patients with APDS, need to be comprehensively analyzed in the future. This approach may have the potential for integrating complex and heterogenous inflammation of bronchial asthma into a clinically meaningful context. We hope that various forms of effective PI3K-δ inhibitors (e.g., inhalation formulation) will soon be available for the treatment of bronchial asthma.
